# Gender Differences in In-Hospital Clinical Outcomes after Percutaneous Coronary Interventions: An Insight from a Japanese Multicenter Registry

**DOI:** 10.1371/journal.pone.0116496

**Published:** 2015-01-30

**Authors:** Yohei Numasawa, Shun Kohsaka, Hiroaki Miyata, Shigetaka Noma, Masahiro Suzuki, Shiro Ishikawa, Iwao Nakamura, Yutaro Nishi, Takahiro Ohki, Koji Negishi, Toshiyuki Takahashi, Keiichi Fukuda

**Affiliations:** 1 Department of Cardiology, Ashikaga Red Cross Hospital, Tochigi, Japan; 2 Department of Cardiology, Keio University School of Medicine, Tokyo, Japan; 3 The University of Tokyo, Healthcare Quality Assessment, Tokyo, Japan; 4 Department of Cardiology, Saiseikai Utsunomiya Hospital, Tochigi, Japan; 5 Department of Cardiology, National Hospital Organization, Saitama National Hospital, Saitama, Japan; 6 Department of Cardiology, Saitama City Hospital, Saitama, Japan; 7 Department of Cardiology, Hino Municipal Hospital, Tokyo, Japan; 8 Department of Cardiology, St Luke’s International Hospital Heart Center, Tokyo, Japan; 9 Department of Cardiology, Tokyo Dental College Ichikawa General Hospital, Chiba, Japan; 10 Department of Cardiology, Yokohama Municipal Hospital, Kanagawa, Japan; S.G.Battista Hospital, ITALY

## Abstract

**Background:**

Gender differences in clinical outcomes after percutaneous coronary intervention (PCI) among different age groups are controversial in the era of drug-eluting stents, especially among the Asian population who are at higher risk for bleeding complications.

**Methods and Results:**

We analyzed data from 10,220 patients who underwent PCI procedures performed at 14 Japanese hospitals from September 2008 to April 2013. A total of 2,106 (20.6%) patients were women. Women were older (72.7±9.7 vs 66.6±10.8 years, p<0.001), and had a lower body mass index (23.4±4.0 vs 24.3±3.5, p<0.001), with a higher prevalence of hypertension (p<0.001), hyperlipidemia (p<0.001), insulin-dependent diabetes (p<0.001), renal failure (p<0.001), and heart failure (p<0.001) compared with men. Men tended to have more bifurcation lesions (p = 0.003) and chronic totally occluded lesions (p<0.001) than women. Crude overall complications (14.8% vs 9.5%, p<0.001) and the rate of bleeding complications (5.3% vs 2.8%, p<0.001) were significantly higher in women than in men. On multivariate analysis in the total cohort, female sex was an independent predictor of overall complications (OR, 1.47; 95% CI, 1.26–1.71; p<0.001) and bleeding complications (OR, 1.74; 95% CI, 1.36–2.24; p<0.001) after adjustment for confounding variables. A similar trend was observed across the middle-aged group (≥55 and <75 years) and old age group (≥75 years).

**Conclusions:**

Women are at higher risk than men for post-procedural complications after PCI, regardless of age.

## Introduction

A high volume of coronary revascularization procedures, such as percutaneous coronary intervention (PCI) is performed for the treatment of coronary artery disease (CAD). Previous studies, mostly performed in Western countries, have described that women fare worse outcomes after PCI [1–10].

Female patients with CAD differ from male patients in terms of age, coronary risk factors, and angiographic characteristics [1–6,11–13]. The patients’ characteristics that are more frequently seen in female compared with male patients include lower body mass index (BMI), older age, a higher prevalence of coronary risk factors, and smaller access and target vessels. These factors are thought to contribute to the gender differences, since they all predispose patients to more complicated procedures [3,5, 10,13,14].

However, gender differences in clinical outcomes after PCI in Asian population have not been thoroughly investigated. The Asian subgroup has been relatively small in previous large-scale registry studies performed in Western countries and few studies regarding the gender differences have been conducted in Asian countries [15,16]. Asian patients with CAD have less traditional coronary risk factors, but have higher rate of bleeding complications after PCI compared with those in Western countries [17,18]. Because the risk profiles of Japanese patients differ from those in Western population, gender differences in clinical outcomes after PCI in Japan need to be investigated.

Furthermore, the gender differences in clinical outcomes after PCI procedures have become controversial in more recent era. The development of newer coronary intervention devices such as smaller sheath, guiding catheters, stents, and balloons has all lead to favorable direction in decreasing the overall complication rate and closing the gender differences [2,6,11,12]. Therefore, in the present study, we examined the gender differences in in-hospital clinical outcomes after modern PCI based on data from a Japanese multicenter registry.

## Material and Methods

### Study Design

The Japan Cardiovascular Database (JCD) is a large, ongoing, prospective multicenter cohort study designed to record clinical background and outcome data for PCI patients in Japan [19–26]. Data for approximately 200 variables is continuously being collected in this study. Participating hospitals are instructed to record data from consecutive hospital visits for PCI and to register these data into an internet-based database system.

Data entered were checked for completeness and internal consistency. Quality assurance of the data was achieved through automatic system validation and reporting of data completeness, education, and training for dedicated clinical research coordinators specifically trained for the present PCI registry. The senior study coordinator (I.U.) and exclusive on-site auditing by investigators (S.K. and H.M.) ensured proper registration of each patient.

PCI with any commercially available coronary device was included. The decision to perform PCI was made according to the investigators’ clinical assessment of the patient. This study does not mandate specific interventional or surgical techniques, such as vascular access, use of specific stents, or closure devices.

Major teaching hospitals within the metropolitan Tokyo area were selected for the pilot phase of this study. Patients were enrolled based on the cardiac event, and all consecutive PCI procedures during the study period were registered, including failure cases. Patients aged <18 years were excluded from the study.

The majority of clinical variables in the JCD are defined according to the National Cardiovascular Data Registry (NCDR). This registry is sponsored by the American College of Cardiology for conducting comparative research to determine factors that can lead to disparities in PCI management. The NCDR is a large PCI registry system with over 1,000,000 entries for ischemic heart disease and over 500,000 entries for PCI collected from more than 500 institutions in the US [7].

### Information disclosure

The study protocol was approved by the institutional review board (IRB) committee at Keio University, School of Medicine in Japan. All the participants provided written informed consent for the present study. Before the launch of the JCD registry, information on the objectives of the present study, its social significance, and an abstract were provided for clinical trial registration with the University Hospital Medical Information Network. This Network is recognized by the International Committee of Medical Journal Editors as an “acceptable registry,” according to a statement issued in September 2004 (UMIN R000005598).

### Study Population

We analyzed data from 10,220 patients who underwent PCI procedures performed at 14 Japanese hospitals participating in the JCD registry from September 2008 to April 2013. Clinical, angiographic, and procedural complications were prospectively entered into the JCD registry database. The choice of access site was based on the preference of the interventional cardiologist. Although the sizes of the sheath and guiding catheter were not protocol-mandated in this cohort, the commonly used size was 6-Fr to 8-Fr in transfemoral intervention, and 6-Fr in transradial intervention (TRI). All patients underwent periprocedural anticoagulation via heparin based on institutional dosing instructions during PCI. A bolus dose of 5,000 to 10,000 IU was initially given and we provided additional doses to keep an activated clotting time level of >300 seconds. We did not have a mandated protocol for hemostasis after the PCI procedures. Details of post-procedural management were left to the primary operators’ decision. The recommended antiplatelet therapy was long-term 81 mg aspirin daily, and a thienopyridine (75 mg clopidogrel or 200 mg ticlopidine daily). Basically, loading dose of clopidogrel was 300 mg, and dual antiplatelet therapy was continued for at least 12 months after DES implantation, and 1 month after bare metal stent implantation.

The endpoints were defined as in-hospital mortality and other complications. Complications were defined as all complications: severe coronary artery dissection or coronary perforation; myocardial infarction after PCI; cardiac shock or heart failure; cerebral bleeding or stroke; and bleeding complications. Severe coronary artery dissection was defined as an intimal tear of coronary artery leading to impaired blood flow (final TIMI flow grade <3) on the angiogram. Myocardial infarction was defined as the new occurrence of a biomarker-positive myocardial infarction after PCI. Bleeding complications in this registry were further defined as those requiring blood transfusion, prolonging hospital stay, or causing a decrease in hemoglobin of >3.0 g/dL. Furthermore, bleeding complications were divided into puncture-site bleeding, retroperitoneal bleeding, gastrointestinal bleeding, genitourinary bleeding, or other bleeding. Hematomas >10 cm for femoral access or > 2cm for radial access also qualified as access site bleeding.

### Data Analysis

Continuous variables are expressed as mean ± standard deviation (SD). Categorical variables are expressed as percentages. Continuous variables were compared using the Student’s t-test, and the differences between categorical variables were examined using the chi-square test.

Multivariate logistic regression analysis was performed to investigate the independent predictors regarding overall and bleeding complications. Variables submitted to the model included age, sex, BMI >30, renal failure, diabetes mellitus, previous heart failure, chronic obstructive pulmonary disease, cardiogenic shock, urgent PCI, emergent PCI, left main disease, three vessel disease, ST elevation myocardial infarction (STEMI), non-STEMI, cardiopulmonary arrest, and TRI. In addition, all patients were stratified into three groups by age; young group (age <55 years: women = 87, men = 1,114), middle-aged group (age ≥55 and <75 years: women = 1,038, men = 4,933), and old age group (age ≥75 years: women = 981, men = 2,067). Multivariate analysis was performed in each group to investigate the relationship between sex and age for overall and bleeding complications.

All statistical calculations and analyses were performed using SPSS version 15 (SPSS, Chicago, IL, USA.). A p value <0.05 was considered statistically significant.

## Results

### Baseline clinical characteristics

The baseline clinical characteristics of the 10,220 patients according to sex are shown in Table 1. Of the 10,220 patients, 2,106 (20.6%) were women. Women were older (p<0.001), had a lower BMI (p<0.001), and a higher prevalence of hypertension (p<0.001), hyperlipidemia (p<0.001), insulin-dependent diabetes mellitus (p<0.001), renal failure, such as chronic kidney disease ≥stage 3 (p<0.001) and hemodialysis (p = 0.015), previous heart failure (p<0.001), non-STEMI (p = 0.034), and unstable angina (p = 0.006) than men. In contrast, Men tended to have a higher prevalence of a smoking habit (p<0.001), peripheral artery disease (p = 0.030), chronic obstructive pulmonary disease (p = 0.006), previous PCI (p<0.001), and previous myocardial infarction (p<0.001) than women. The rate of administration of antiplatelet agents was not different between sexes.

**Table 1:** Baseline Clinical Characteristics in Women and Men.

	**Women (n = 2106)**	**Men (n = 8114)**	**p Value**
Age, years	72.7 ± 9.7	66.6 ± 10.8	<0.001
Height (cm)	150.2 ± 6.2	165.1 ± 6.6	<0.001
Weight (kg)	53.0 ± 10.2	66.6 ± 11.6	<0.001
BMI (kg/m^2^)	23.4 ± 4.0	24.3 ± 3.5	<0.001
Coronary risk factors			
Hypertension	1629(77.4%)	5924(73.0%)	<0.001
Hyperlipidemia	1435(68.1%)	5309(65.4%)	<0.001
Diabetes mellitus	865(41.1%)	3421(42.2%)	0.372
Insulin use	243(11.5%)	649(8.0%)	<0.001
Current smoking	300(14.2%)	3257(40.1%)	<0.001
Previous PCI	686(32.6%)	3016(37.2%)	<0.001
Previous CABG	114(5.4%)	435(5.4%)	0.917
Previous HF	243(11.5%)	656(8.1%)	<0.001
Previous MI	461(21.9%)	2114(26.1%)	<0.001
CVD	180(8.5%)	747(9.2%)	0.371
PAD	147(7.0%)	685(8.4%)	0.030
COPD	45(2.1%)	268(3.3%)	0.006
STEMI	528(25.1%)	1957(24.1%)	0.362
non-STEMI	194(9.2%)	631(7.8%)	0.034
Unstable angina	427(20.3%)	1431(17.6%)	0.006
Stable angina	596(28.3%)	2184(26.9%)	0.206
Cardiogenic shock	87(4.1%)	346(4.3%)	0.851
Cardiopulmonary arrest	38(1.8%)	241(3.0%)	0.003
Emergent PCI	516(24.5%)	1807(22.3%)	0.030
Urgent PCI	478(22.7%)	1655(20.4%)	0.022
CKD ≥ stage 3	478(22.7%)	1171(14.4%)	<0.001
Hemodialysis	111(5.3%)	328(4.0%)	0.015
Antiplatelet agents			
Aspirin	2023(96.1%)	7814(96.3%)	0.411
Clopidogrel	1555(73.8%)	6051(74.6%)	0.428
Ticlopidine	54(2.6%)	288(3.5%)	0.108
Cirostazol	36(1.7%)	136(1.7%)	0.752

Values are presented as n (%) or mean ± SD, as indicated.

BMI = body mass index; PCI = percutaneous coronary intervention; CABG = coronary artery bypass grafting; HF = heart failure; MI = myocardial infarction; CVD = cerebrovascular disease; PAD = peripheral artery disease; COPD = chronic obstructive pulmonary disease; STEMI = ST elevation myocardial infarction; CKD = chronic kidney disase.

### Angiographic and Procedural Data

Angiographic and procedural data are shown in Table 2. Men tended to have more bifurcation lesions (p = 0.003) and chronic totally occluded lesions (p<0.001) than women. Overall, approximately one-third of the patients underwent TRI, although this was less frequent in women than in men (p<0.001). Bare metal stents (p<0.001) and intravascular ultrasound (p = 0.016) were used more frequently in men than in women. Plain old balloon angioplasty was performed more frequently in women than in men (p = 0.003).

**Table 2:** Angiographical and Procedural Data in Women and Men.

	**Women (n = 2106)**	**Men (n = 8114)**	**p Value**
CAD stenosis (≥50%)			
2 vessel disease	921(43.7%)	3616(44.6%)	0.506
3 vessel disease	514(24.4%)	1992(24.6%)	0.908
Bifurcation lesion	496(23.6%)	2171(26.8%)	0.003
LMT lesion	186(8.8%)	780(9.6%)	0.296
CTO lesion	90(4.3%)	554(6.8%)	<0.001
Type C lesion	592(28.1%)	2387(29.4%)	0.247
Transradial Intervention	544(25.8%)	2646(32.6%)	<0.001
Transfemoral Intervention	1499(71.2%)	5267(64.9%)	<0.001
Drug eluting stent	1368(65.0%)	5130(63.2%)	0.148
Bare metal stent	399(18.9%)	1898(23.4%)	<0.001
POBA	184(8.7%)	553(6.8%)	0.003
IVUS use	686(32.6%)	2873(35.4%)	0.016
IABP use (pre PCI)	36(1.7%)	155(1.9%)	0.584
Closure device	286(13.6%)	1042(12.8%)	0.364
X-ray time (min)	29.6 ± 21.4	29.8 ± 21.6	0.770

Values are presented as n (%) or mean ± SD, as indicated.

CAD = coronary artery disease; LMT = left main trunk; CTO = chronic total occlusion; POBA = plain old angioplasty; IVUS = intravascular ultrasound; IABP = intra-aortic balloon pumping; PCI = percutaneous coronary intervention.

### Complications

In-hospital outcomes in the overall population are shown in Table 3. Women had higher crude complication rates than men (p<0.001), although the in-hospital mortality rate was not different between sexes. Severe coronary artery dissection (p<0.001), heart failure after PCI (p = 0.009), and cerebral infarction (p = 0.003) were observed more frequently in women than in men. In addition, the rate of bleeding complications within 72 hours (p<0.001), including puncture site bleeding (p = 0.001), puncture site hematoma (p<0.001), and the rate of receiving blood transfusion (p<0.001) were significantly higher in women than in men.

**Table 3:** Complications in Women and Men.

	**Women (n = 2106)**	**Men (n = 8114)**	**p Value**
Total complications	312(14.8%)	772(9.5%)	<0.001
In-hospital mortality	57(2.7%)	208(2.6%)	0.706
Severe coronary artery dissection	42(2.0%)	83(1.0%)	<0.001
Coronary perforation	22(1.0%)	79(1.0%)	0.814
Myocardial infarction after PCI	52(2.5%)	174(2.1%)	0.362
Heart failure after PCI	57 (2.7%)	145 (1.8%)	0.009
Cardiac shock	52(2.5%)	161(2.0%)	0.169
Cardiac tamponade	11(0.5%)	21(0.3%)	0.076
Cerebral bleeding	2(0.1%)	5(0.1%)	0.639
Cerebral infarction	17(0.8%)	25(0.3%)	0.003
Bleeding complications within 72 hours	111(5.3%)	225(2.8%)	<0.001
Puncture site bleeding	39(1.9%)	78(1.0%)	0.001
Puncture site hematoma	41(1.9%)	58(0.7%)	<0.001
Retroperitoneal bleeding	6(0.3%)	9(0.1%)	0.100
Gastrointestinal bleeding	12(0.6%)	31(0.4%)	0.256
Genitourinary bleeding	2(0.1%)	15(0.2%)	0.551
Other bleeding	31(1.5%)	77(0.9%)	0.038
Blood transfusion	110(5.2%)	169(2.1%)	<0.001
New Hemodialysis	22(1.0%)	81(1.0%)	0.817

Values are presented as n (%).

PCI = percutaneous coronary intervention.

The results of multivariate logistic regression analysis on overall and bleeding complications are shown in Table 4 and 5. Importantly, female sex was an independent predictor of overall complications after adjustment for possible confounding variables in the total cohort (odds ratio [OR], 1.47; 95% confidence interval [CI], 1.26–1.71; p<0.001), the middle-aged group (OR, 1.43; 95% CI, 1.14–1.79; p = 0.002), and the old age group (OR, 1.47; 95% CI, 1.17–1.84; p = 0.001). Furthermore, female sex was also an independent predictor of bleeding complications in the total cohort (OR, 1.74; 95% CI, 1.36–2.24; p<0.001), the middle-aged group (OR, 2.09; 95% CI, 1.44–3.03; p<0.001), and the old age group (OR, 1.64; 95% CI, 1.15–2.34; p = 0.006). Because the number of female patients in the young group (age <55 years) was limited (87/10,220, 0.8%), multivariate analysis in this group was not performed. Other variables that were independent predictors for overall complications in all of the groups stratified by age included renal failure, cardiogenic shock, previous heart failure, left main disease, and cardiopulmonary arrest. Notably, TRI was an independent predictor of preventing overall and bleeding complications across all of the age groups.

**Table 4:** Multivariate Logistic Regression Analysis on Overall Complications.

	**Total cohort (Women = 2106, Men = 8114)**			**Age 55–74 years (Women = 1038, Men = 4933)**			**Age ≥ 75 years (Women = 981, Men = 2067)**		
	OR	95% CI	p Value	OR	95% CI	p Value	OR	95% CI	p Value
Female sex	1.47	1.26–1.71	<0.001	1.43	1.14–1.79	0.002	1.47	1.17–1.84	0.001
Age	1.02	1.01–1.03	<0.001	1.04	1.02–1.05	<0.001	1.01	0.99–1.04	0.288
BMI >30	1.04	0.77–1.40	0.812	0.99	0.66–1.51	0.996	0.84	0.38–1.87	0.676
Renal failure	2.14	1.71–2.67	<0.001	2.29	1.69–3.10	<0.001	2.24	1.58–3.19	<0.001
Cardiogenic shock	2.25	1.70–3.00	<0.001	2.42	1.58–3.71	<0.001	1.71	1.10–2.64	0.016
Previous heart failure	1.50	1.22–1.85	<0.001	1.41	1.03–1.94	0.034	1.56	1.16–2.08	0.003
Left main disease	1.54	1.26–1.88	<0.001	1.71	1.29–2.26	<0.001	1.44	1.07–1.93	0.015
STEMI	1.53	1.20–1.95	0.001	1.69	1.20–2.39	0.003	1.33	0.92–1.94	0.133
non-STEMI	1.20	0.92–1.57	0.182	1.41	0.96–2.07	0.079	1.03	0.68–1.55	0.898
Cardiopulmonary arrest	1.92	1.37–2.69	<0.001	1.77	1.10–2.86	0.020	2.14	1.14–4.01	0.018
Transradial Intervention	0.65	0.54–0.77	<0.001	0.58	0.45–0.74	<0.001	0.71	0.54–0.93	0.013

OR = odds ratio; CI = confidence interval; BMI = body mass index; STEMI = ST elevation myocardial infarction.

**Table 5:** Multivariate Logistic Regression Analysis on Bleeding Complications Within 72 Hours.

	**Total cohort (Women = 2106, Men = 8114)**			**Age 55–74 years (Women = 1038, Men = 4933)**			**Age ≥ 75 years (Women = 981, Men = 2067)**		
	OR	95% CI	p Value	OR	95% CI	p Value	OR	95% CI	p Value
Female sex	1.74	1.36–2.24	<0.001	2.09	1.44–3.03	<0.001	1.64	1.15–2.34	0.006
Age	1.06	1.01–1.03	<0.001	1.03	1.01–1.07	0.023	0.97	0.92–1.01	0.164
BMI >30	0.80	0.45–1.41	0.453	0.68	0.29–1.60	0.383	1.17	0.40–3.40	0.768
Renal failure	2.60	1.87–3.63	<0.001	2.88	1.78–4.67	<0.001	2.79	1.72–4.54	<0.001
Cardiogenic shock	1.88	1.21–2.93	0.005	3.28	1.74–6.19	<0.001	0.65	0.28–1.51	0.327
Previous heart failure	1.54	1.10–2.15	0.011	1.39	0.80–2.40	0.236	1.59	1.02–2.47	0.039
Left main disease	1.43	1.03–1.99	0.029	1.75	1.08–2.81	0.021	1.19	0.74–1.92	0.462
STEMI	1.51	1.01–2.28	0.047	1.53	0.81–2.87	0.181	1.51	0.67–2.26	0.496
non-STEMI	2.07	1.37–3.12	0.001	2.46	1.31–4.61	0.005	1.81	1.00–3.29	0.049
Cardiopulmonary arrest	2.56	1.57–4.18	<0.001	1.84	0.93–3.62	0.076	2.89	1.05–7.92	0.039
Transradial Intervention	0.51	0.36–0.70	<0.001	0.45	0.27–0.75	0.002	0.51	0.31–0.82	0.006

OR = odds ratio; CI = confidence interval; BMI = body mass index; STEMI = ST elevation myocardial infarction.

## Discussion

The major findings of this study showed gender differences among patients with CAD who underwent PCI, in one of the largest contemporary multicenter registries in Japan. Even after adjustment for differences in baseline clinical characteristics, women had worse in-hospital complications during and after PCI than men, especially for bleeding and vascular complications across all of the age groups. Clinical trials are generally faced with the difficulty of having a very high proportion of male patients, especially in Asia. Our dataset included more than 10,000 patients and about one-fifth was female. This high percentage of female patients allowed us to attempt to analyze the various in-hospital outcomes between male and female patients across all the age groups.

Female patients with CAD differ from male patients. Many previous studies have reported that women who underwent PCI were older, and had a higher prevalence of coronary risk factors including hypertension, diabetes mellitus, and renal insufficiency than men [1–6,11,12]. Women also have a higher rate of bleeding complications than men [1–3,5–10]. In our present study, although men tended to have more complex lesions including bifurcation lesions (p = 0.003) and chronic totally occluded lesions (p<0.001) than women, female sex was an independent predictor of overall complications (OR, 1.47; 95% CI, 1.26–1.71; p<0.001) and bleeding complications (OR, 1.74; 95% CI, 1.36–2.24; p<0.001) after adjustment for confounding variables.

Japan, albeit a small country, performs approximately 200,000 PCI procedures annually, and primary PCI facilities are available throughout the country owing to the socialized medical system. This situation enables more timely access to revascularization in high-risk patients with acute coronary syndrome. Moreover, patients with CAD in Japan have different characteristics compared with Western patients (e.g., older, smoke more frequently, and have less traditional risk factors, except for diabetes), tend to have more bleeding complications during and after PCI [17,18], and undergo complex procedures. Despite these differences, our study results are consistent with reports from Western countries regarding these factors.

Few prior studies have examined the gender differences in clinical outcomes after PCI in Asian population. Woo et al. reported that adverse in-hospital post-PCI cardiovascular events occurred more often in female patients than in male patients in a Chinese single center study [15]. Shin et al. reported that female patients had a tendency for more frequent bleeding complications than male patients after PCI with transradial approach in a Korean TRI registry [16]. In our present study, we also suggest that there is an important “gender gap” in Japanese patients who are older and have a lower BMI compared with patients in Western countries. Furthermore, owing to the large number of patients included in our registry, we were able to perform separate analyses on three different age groups. And from this, women still were found to be at higher risk in each of the subgroups compared with men.

Potential overdosing of antiplatelets or anticoagulants, and differences in platelet biology have been reported as reasons for the higher bleeding risk in women. This may be particularly true for the lean Asian population [8,13,27]. Glomerular filtration rate is based on the patient’s weight, and tends to be relatively lower in women than in men, despite the same levels of serum creatinine; lower renal function may affect pharmacokinetic differences between sexes. Alexander et al. reported that women are more likely to receive excess doses of antithrombotic agents, and excess doses of glycoprotein IIb/IIIa inhibitors are associated with an increased risk of bleeding complications, especially in women [8,27]. Although glycoprotein IIb/IIIa inhibitors are not available in Japan, similar pharmacological effects may be observed for other antiplatelets and anticoagulants [8,10,27]. Notably, almost all of our patients underwent PCI with a unified regimen of aspirin, clopidogrel, and heparin during the procedure. This is because other agents, such as prasugrel, ticagrelor, and low-molecular weight heparin had not been approved for use in patients with CAD in Japan at the time the study was conducted. The unified regimen of antiplatelets and anticoaglants for both men and women may affect the gender differences in bleeding complications after PCI in Japan.

Younger women have a higher relative risk of worse outcomes, including bleeding complications than age-matched men after PCI [4,5,28–30]. In our study, to investigate the relationship between sex and age, all patients were stratified into three groups by age, and we performed multivariate logistic regression analysis for overall and bleeding complications in each group, except for the youngest group (age <55 years) because of an insufficient number of young female patients (0.8%). Across all ages, female sex was an independent predictor of overall and bleeding complications. While the precise mechanism is not well understood, sex hormone levels may affect gender differences and sex-age interactions in women on outcome after PCI [5,30]. Although the OR for bleeding complications was slightly higher in the middle-aged group than in the old age group, its clinical implication is unclear. Notably, only a few premenopausal women underwent PCI in Japan. The proportion of young women in the total cohort in our study was lower than that in previous studies [5,28,30]. More aggressive screening of CAD, including risk factors in young women should be considered.

Although the overall incidence of bleeding complications related to PCI has been decreasing [3,13], it still remains one of the most important challenges in modern PCI. Bleeding complications are associated with an increased risk of adverse events, including mortality [3,31–33]. Therefore, efforts for reducing bleeding complications are important. The transradial approach to PCI has been implicated recently in reducing bleeding complications compared with conventional transfemoral intervention [9,23,34,35]. In fact, approximately one-third of all of the PCIs in our dataset were performed with transradial approach, and this is consistent with other Japanese studies [23]. Traditionally, TRI has been performed less frequently in women than in men because of a smaller radial artery diameter [9], as observed in our study. Given the higher complication rate, particularly hemorrhage-related complications, use of TRI may aid in decreasing these events, especially in women [1,3,9]. In our study, TRI was an independent predictor of preventing overall and bleeding complications in multivariate logistic regression analysis across all ages (Table 4 and 5). Although TRI is more commonly performed in Japan than in Western countries [23], more frequent use of radial access in women for reducing bleeding complications should be considered.

Anatomically, women have a smaller body size and smaller arteries than men [36]. Smaller vessels may contribute to higher rates of bleeding complications and vascular injury, including coronary artery dissection, as observed in our study [5,14]. Another important reason for the increased rates of vascular events in female patients is more vulnerable endothelial layer of blood vessels compared with male patients [5]. Most coronary artery dissections can be treated by stenting, but this sometimes results in a serious condition with slow flow or no flow in the injured coronary artery. Therefore, women are also at greater risk of vascular injury during PCI. In addition, a smaller coronary artery size in women might be one of the major reasons that PCI without stents (plain old balloon angioplasty) was performed more frequently in women than in men in our study. Because a small size (2.25 mm) of DES is currently available, PCI for small vessels with small stents are becoming more popular, especially in women. Endovascular techniques and devices have evolved over the years. Smaller sheaths, guiding catheters, stents, and balloons are available, and women may benefit from these.

### Study limitations

The first limitation is that this study was an observational clinical trial. Although we performed multivariate logistic regression analysis to adjust for possible confounding variables, some selection bias might not have been completely adjusted for in our statistical model. Another limitation is that the size of the sheaths and guiding catheters was not recorded in our registry. This factor might be associated with bleeding complication rates. Finally, the impact of sex and in-hospital bleeding complications on long-term clinical outcomes among patients who underwent PCI should be investigated in our registry in the future.

### Conclusions

While gender differences in outcomes after PCI procedures have been decreasing in the modern DES era, we should continue to recognize that women are still at greater risk for complications during and after PCI, including coronary artery dissection, heart failure, and bleeding complications. Use of TRI may aid in reducing the rate of complications after PCI, especially bleeding complications.
